# Genetic approaches to elucidating cortical and hippocampal GABAergic interneuron diversity

**DOI:** 10.3389/fncel.2024.1414955

**Published:** 2024-07-24

**Authors:** Robert Machold, Bernardo Rudy

**Affiliations:** ^1^Neuroscience Institute, New York University Grossman School of Medicine, New York, NY, United States; ^2^Department of Neuroscience and Physiology, New York University Grossman School of Medicine, New York, NY, United States; ^3^Department of Anesthesiology, Perioperative Care and Pain Medicine, New York University Grossman School of Medicine, New York, NY, United States

**Keywords:** GABAergic interneurons, intersectional genetics, transgenic, cortex, hippocampus, subtypes

## Abstract

GABAergic interneurons (INs) in the mammalian forebrain represent a diverse population of cells that provide specialized forms of local inhibition to regulate neural circuit activity. Over the last few decades, the development of a palette of genetic tools along with the generation of single-cell transcriptomic data has begun to reveal the molecular basis of IN diversity, thereby providing deep insights into how different IN subtypes function in the forebrain. In this review, we outline the emerging picture of cortical and hippocampal IN speciation as defined by transcriptomics and developmental origin and summarize the genetic strategies that have been utilized to target specific IN subtypes, along with the technical considerations inherent to each approach. Collectively, these methods have greatly facilitated our understanding of how IN subtypes regulate forebrain circuitry via cell type and compartment-specific inhibition and thus have illuminated a path toward potential therapeutic interventions for a variety of neurocognitive disorders.

## Introduction

The marvelous diversity of locally projecting GABAergic inhibitory interneurons (INs) has been appreciated for over a century, beginning with the detailed morphological observations of Ramón y Cajal. In recent years, our understanding of the molecular and circuit specialization of cortical and hippocampal INs has increased dramatically, and there are a number of excellent reviews to recommend on this subject (Klausberger and Somogyi, 2008; Tremblay et al., 2016; Bandler et al., 2017; Pelkey et al., 2017; Wamsley and Fishell, 2017; Feldmeyer et al., 2018; Lim et al., 2018; Huang and Paul, 2019; Fishell and Kepecs, 2020; Gutman-Wei and Brown, 2021; Kullander and Topolnik, 2021; Topolnik and Tamboli, 2022; Kessaris and Denaxa, 2023). The discovery of molecular markers corresponding to the distinct electrophysiological and morphological properties of IN subpopulations has greatly facilitated work on elucidating IN subtype functionality (Kawaguchi and Kubota, 1996; Kawaguchi and Kubota, 1997; Kubota et al., 2011; Rudy et al., 2011; Taniguchi et al., 2011; Pfeffer et al., 2013; He et al., 2016; Gouwens et al., 2020; Bugeon et al., 2022) and has guided the implementation of genetic strategies to experimentally target and manipulate molecularly defined cell subtypes (Urban and Rossier, 2012; Huang et al., 2014; Taniguchi, 2014; He and Huang, 2018; Hanson and Wester, 2022). Over the last few decades, a remarkable toolkit for genetic targeting of cell populations in the mouse has been developed, including transgenic and knock-in approaches to express recombinases (e.g., Cre or Flp) under the control of specific marker genes (driver lines), as well as reporter lines to express fluorescent proteins or other actuators in response to recombinase activity (Madisen et al., 2010, 2012, 2015; Taniguchi et al., 2011; He et al., 2016; Daigle et al., 2018). In parallel, there has been a revolution in recombinant AAV (rAAV)-based viral vectors to target IN cell populations, including recombinase-dependent constructs (e.g., AAV-DIO) as well as the ongoing discovery and implementation of cell type-specific short promoters in mouse and other species (Dimidschstein et al., 2016; Haery et al., 2019; Hrvatin et al., 2019; Mehta et al., 2019; Nair et al., 2020; Vormstein-Schneider et al., 2020; Duba-Kiss et al., 2021; Graybuck et al., 2021; Hoshino et al., 2021; Mich et al., 2021; Challis et al., 2022; Pouchelon et al., 2022; Campos et al., 2023; Niibori et al., 2023).

**Figure 1:**
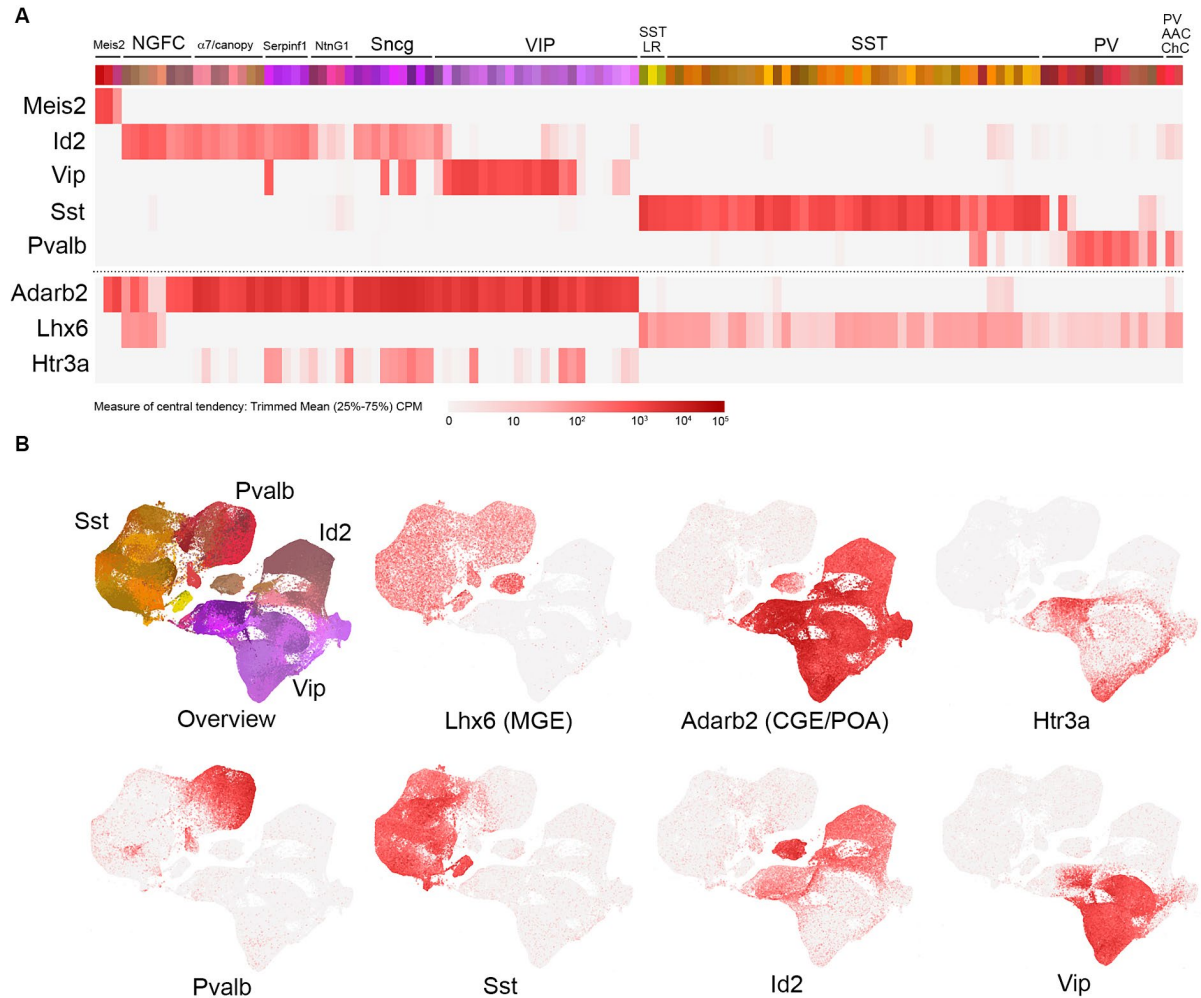
Overview of cortical and hippocampal IN transcriptomic subtypes. **(A)** Schematic view of the total GABAergic IN scRNAseq data from Yao et al. (2021) [portal.brain-map.org/atlases-and-data/rnaseq; whole cortex and hippocampus—10x genomics (2020) with 10x smart-seq taxonomy (2021)]. Each colored bin represents a subpopulation of cells clustered as described by Yao et al. (2021). Groups of related bins are annotated here based on gene expression and putative cell type assignment, with heatmaps of the primary gene marker mRNA levels represented as trimmed means (counts per million: CPM, with color intensity reflecting log2 scale as indicated) aligned below. These primary markers are as follows(from left to right): Meis2, Id2, Vip, Sst, and Pvalb. The Id2 IN population encompasses neurogliaform cells (NGFCs), α7/canopy cells, Serpinf1, NtnG1, and Sncg subtypes (detailed in Figure 2). The VIP group is represented as one group here, with details elaborated in Figure 3. The Sst group is represented as two main groups: Sst long-range (Sst LR) and other Sst subtypes (detailed in Figure 4). Likewise, the Pvalb group is represented as PV and PV axo-axonic/chandelier (PV AAC/ChC) subtypes (detailed in Figure 5). To complement these primary IN markers, heatmaps for Adarb2 (CGE/POA) and Lhx6 (MGE) are shown, along with Htr3a. **(B)** Scatterplots of the total IN population from Yao et al. (2021) illustrate the transcriptomic diversity of IN subtypes in reduced dimensional space. The color scheme of the bins in A is maintained in the cell representations shown in the overview, and the general locations of the four main IN groups (Id2, Vip, Sst, and Pvalb) are indicated. Individual scatterplots for Lhx6 (MGE), Adarb2 (CGE/POA), Htr3a, Pvalb, Sst, Id2, and Vip are shown, with the red color intensity reflecting the trimmed mean CPM values (log2 scale) as in **(A)**.

The emergence of technologies to evaluate single-cell transcriptomes (scRNAseq) has revolutionized our understanding of molecular cell type heterogeneity and, in particular, has provided deep insights into mouse forebrain GABAergic IN subtype diversity (Zeisel et al., 2015; Tasic et al., 2016; Paul et al., 2017; Harris et al., 2018; Munoz-Manchado et al., 2018; Saunders et al., 2018; Tasic et al., 2018; Gouwens et al., 2020; Yao et al., 2021). In this review, we present an exposition of the transcriptomic analysis of cortical and hippocampal INs published recently by the Allen Institute (Yao et al., 2021; portal.brain-map.org/atlases-and-data/rnaseq; 10x genomics with 10x smart-seq taxonomy). This extensive dataset, comprised of scRNAseq profiles from roughly 170,000 curated INs clustered into 123 bins, provides an exceptionally high-resolution view of IN transcriptomic identity and serves as a useful framework for the discussion of subtype-specific genetic targeting strategies (Figure 1). Along this framework, we have aligned individual heatmaps of gene expression, with transcript levels represented as color intensity corresponding to trimmed mean (25–75%) counts per million (CPM) on a log2 scale.^1^ From a bird eye view, this approach illustrates the main contours of IN subtype diversity, with five primary markers covering the vast majority of INs: Meis2, Id2, Vip, Sst, and Pvalb (Figure 1; see abbreviations list at the end of the manuscript). The relative abundance of each primary IN group varies across different cortical/hippocampal areas (Kim et al., 2017; Yao et al., 2021), but in somatosensory barrel field cortex, the proportions are approximately Id2 (18%), VIP (12%), Sst (30%), and Pvalb (40%) (Rudy et al., 2011; Tremblay et al., 2016; Machold et al., 2023). We have also included scatterplots (see footnote 1) of the overall IN landscape to highlight the locales of selected IN subtypes. It is important to note that the study by Yao et al. (2021) includes data on the proportions of each individual IN subtype across different cortical and hippocampal areas, as well as a mapping of the previous transcriptomic clusters described in a study by Tasic et al. (2018) to the bins in this expanded dataset.

**Figure 2:**
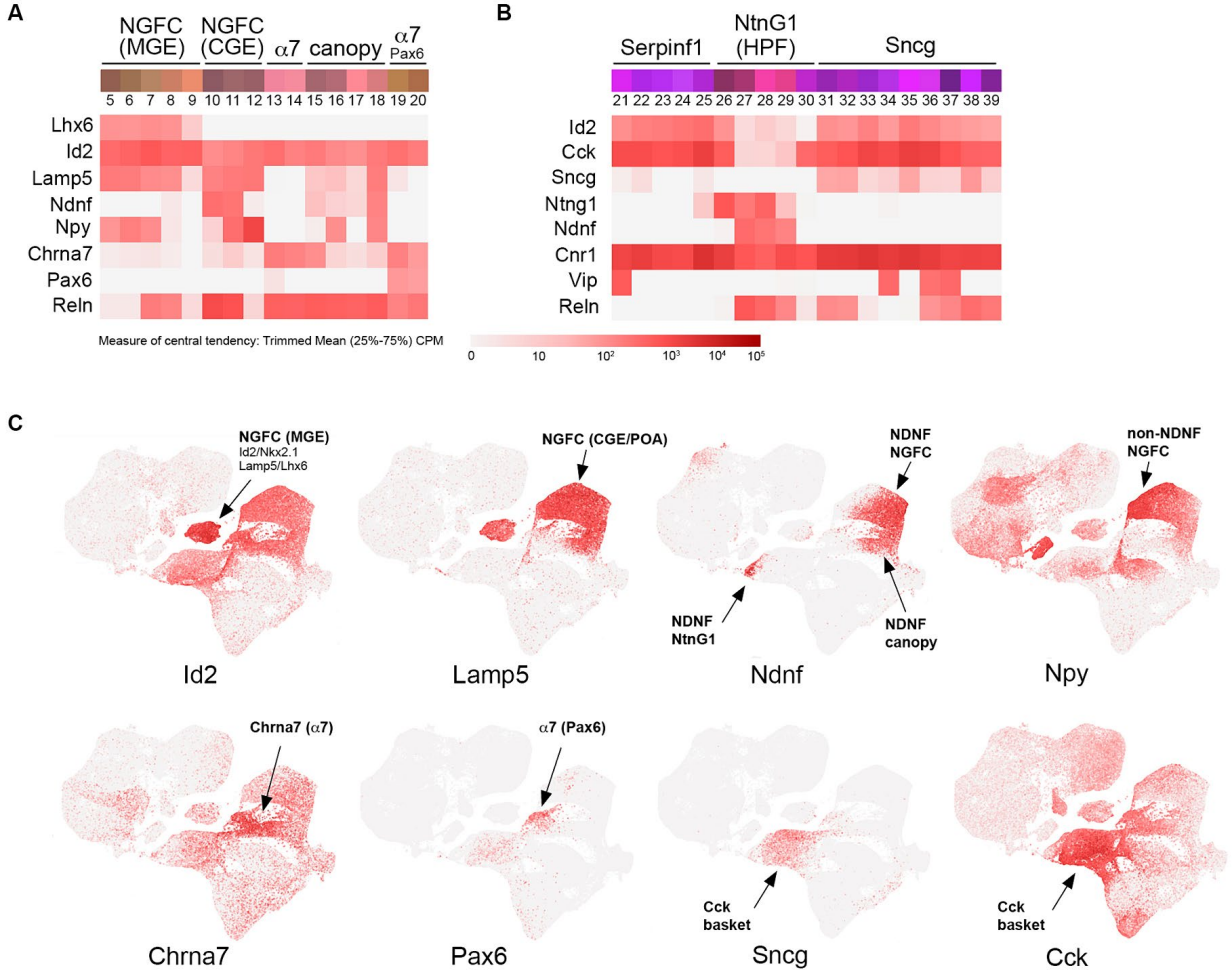
Id2-Lamp5-Sncg transcriptomic subtypes. **(A)** INs corresponding to MGE-derived NGFC (Lhx6+; bins 5–9), CGE-derived NGFC (non-Lhx6+; bins 10–12), α7 cells (bins 13–14 and Pax6+ bins 19–20), and canopy cells (bins 15–18). Heatmaps for Lhx6, Id2, Lamp5, Ndnf, Npy, Chrna7, Pax6, and Reln transcripts are aligned below (trimmed mean CPM in each bin is represented by red color intensity on a log2 scale, as indicated by the guide located below the heatmaps). **(B)** INs corresponding to Serpinf1 (bins 21–25), NtnG1 (bins 26–30), and Sncg (bins 31–39) cells. Heatmaps for Id2, Cck, Sncg, NtnG1, Ndnf, Cnr1, Vip, and Reln are aligned below. **(C)** Individual scatterplots for Id2, Lamp5, Ndnf, Npy, Chrna7, Pax6, Sncg, and Cck are shown, with annotation and arrows highlighting the approximate location of the indicated IN subtypes. HPF, hippocampal formation.

**Figure 3:**
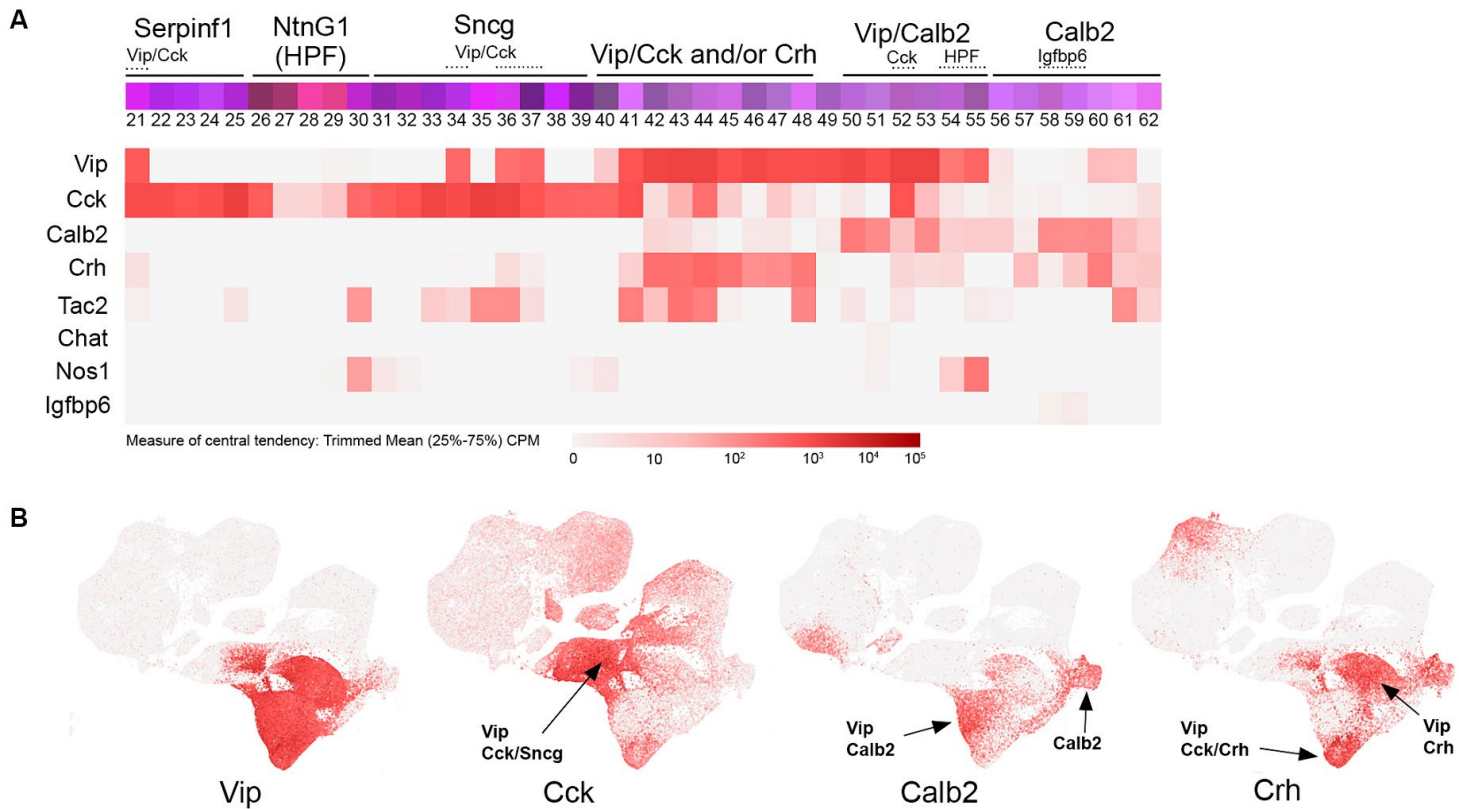
VIP transcriptomic subtypes. **(A)** Overview of VIP IN transcriptomic diversity, with heatmaps for Vip, Cck, Calb2, Crh, Tac2, Chat, Nos1, and Igfbp6 transcripts shown below (trimmed mean CPM in each bin is represented by red color intensity on a log2 scale, as indicated by the guide located below the heatmaps). Several subgroups of VIP INs are annotated above the colored bins, including Vip/Cck, Vip/Crh, Vip/Calb2, and Calb2 (non-VIP) cells. **(B)** Individual scatterplots for Vip, Cck, Calb2, and Crh are shown, with annotation and arrows highlighting the approximate location of the indicated IN subtypes. HPF, hippocampal formation.

**Figure 4:**
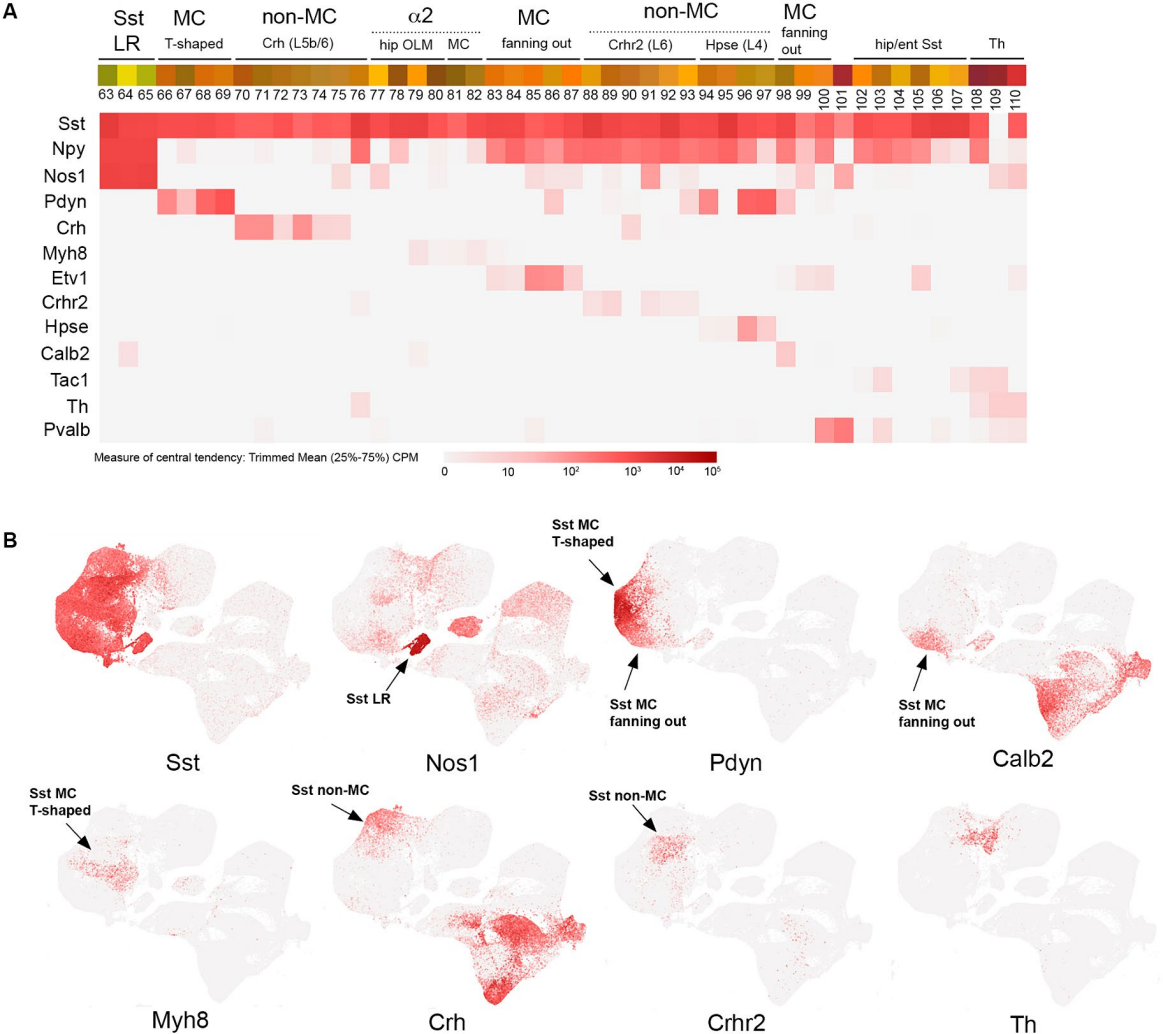
SST transcriptomic subtypes. **(A)** Overview of SST IN transcriptomic diversity, with heatmaps for Sst, Npy, Nos1, Pdyn, Crh, Myh8, Etv1, Crhr2, Hpse, Calb2, Tac1, Th, and Pvalb transcripts shown below (trimmed mean CPM in each bin is represented by red color intensity on a log2 scale, as indicated by the guide located below the heatmaps). Different subgroups of SST INs are annotated above the colored bins, including Sst LR (long range), MC (Martinotti), non-MC (non-Martinotti), α2 (Chrna2-expressing OLM and MC cells), hippocampal/entorhinal Sst cells, and Th cells. **(B)** Individual scatterplots for Sst, Nos1, Pdyn, Calb2, Myh8, Crh, Crhr2, and Th are shown, with annotation and arrows highlighting the approximate location of the indicated IN subtypes.

**Figure 5:**
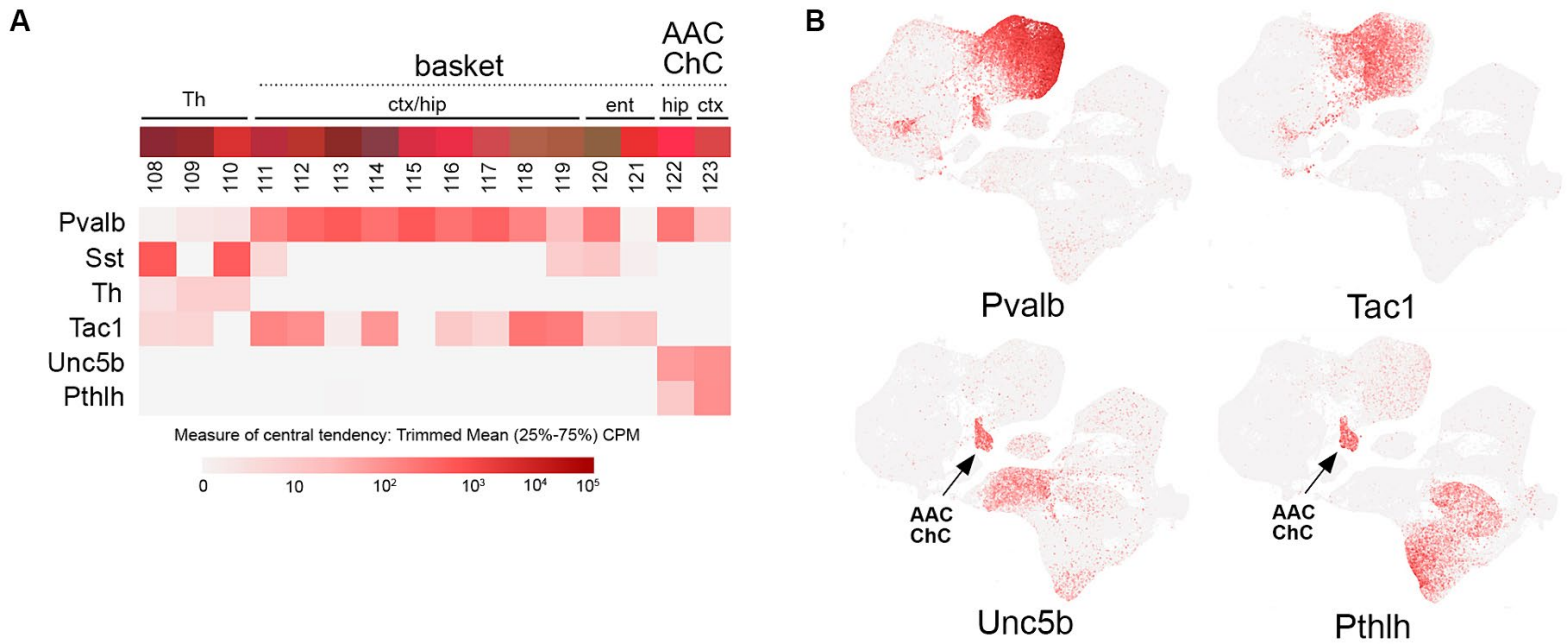
PV transcriptomic subtypes. **(A)** Overview of PV (Pvalb) IN transcriptomic diversity, with heatmaps for Pvalb, Sst, Th, Tac1, Unc5b, and Pthlh transcripts shown below (trimmed mean CPM in each bin is represented by red color intensity on a log2 scale, as indicated by the guide located below the heatmaps). The two main groups of PV INs (basket; bins 111–119, and axo-axonic/chandelier; bins 122–123) are annotated above the colored bins. **(B)** Individual scatterplots for Pvalb, Tac1, Unc5b, and Pthlh are shown, with annotation and arrows highlighting the axo-axonic/chandelier (AAC/ChC) cluster.

Within several of these primary marker groups, we have delineated specific subpopulations that are fundamentally distinct based on their transcriptomic properties as well as other features described in the literature. For example, the Id2 group (Mayer et al., 2018; Machold et al., 2023) encompasses neurogliaform cells (NGFC), α7/canopy cells (Schuman et al., 2019), Serpinf1 cells (a subset of CCK+ INs; Tasic et al., 2018), NtnG1 cells (a hippocampal-specific population that expresses NDNF; see Figure 2), and Sncg cells (CCK+ basket cells). VIP cells here are represented as one main group, with some of the VIP cells clustering in the Serpinf1 and Sncg groups (see Figure 3 for further details). The SST INs exhibit extensive diversity overall (see Figure 4), but a primary distinction is between the SST long range (SST LR) subtype and the other SST INs. Likewise, PV INs can be initially delineated into PV (basket type) and PV axo-axonic (AAC) or chandelier cells (ChC; see Figure 5). Note that while these primary markers are reasonably thorough in tiling the overall IN population, there are notable areas of some overlap (e.g., PV/SST; Figures 4, 5), as well as certain minor populations that do not appear to express any of the markers (e.g., Igfbp6 Calb2 INs within the VIP group; see Figure 3).

## Developmental origins

Cortical, hippocampal, and striatal INs arise during embryonic development from two primary germinal zones in the ventral telencephalon, namely, the medial ganglionic eminence (MGE) and the caudal ganglionic eminence (CGE), and undergo an extensive migration to reach their final locations (Bandler et al., 2017; Hu et al., 2017; Wamsley and Fishell, 2017; Lim et al., 2018; Llorca and Deogracias, 2022; Bandler and Mayer, 2023; Kessaris and Denaxa, 2023). All INs are specified from progenitors that express Ascl1 (Mash1) during neurogenesis (Casarosa et al., 1999) and acquire their GABAergic IN identity via expression of a cascade of Dlx homeobox transcription factors (Anderson et al., 1997, 1999; Eisenstat et al., 1999; Stuhmer et al., 2002). The Dlx genes (Dlx1 and 2, and Dlx5 and 6) are organized into two genetic loci, with each pair sharing an intergenic enhancer that was found to be sufficient in transgenic mice to drive expression of reporters or recombinases in newly born INs (Zerucha et al., 2000; Ghanem et al., 2003, 2007). Thus, all INs can be labeled developmentally using Dlx5/6-Cre (Dlx6a-Cre) (Monory et al., 2006) or Dlx5/6-Flpe (Miyoshi et al., 2010) transgenic drivers in combination with reporter lines. More recently, a number of pan-GABAergic knock-in drivers have been made that allow for targeting of all cortical and hippocampal INs, for example, Slc32a1-Cre (VGAT-Cre) (Vong et al., 2011), Slc32a1-Flpo (Vgat-Flpo) (Daigle et al., 2018), and Gad2-Cre (Taniguchi et al., 2011). Of particular interest, rAAV constructs utilizing promoters based on the Dlx intergenic enhancers have been shown to be effective in targeting INs from development through adult ages, both in rodents and other species including non-human primates (Dimidschstein et al., 2016). Other broad GABAergic-specific promoters recently shown to function in rAAVs include mGAD65a (Hoshino et al., 2021). Given the potential translational utility of IN-specific viral vectors, this is an area of exciting ongoing innovation (Duba-Kiss et al., 2021; Campos et al., 2023).

INs originating from the MGE include all PV and SST subtypes, as well as a subset of Id2 NGFC; these MGE lineages all arise from progenitors that express the homeobox transcription factor Nkx2.1 (Sussel et al., 1999). While most cortical and hippocampal MGE INs rapidly downregulate Nkx2.1 expression immediately following their specification (Marin et al., 2000), they maintain expression of Lhx6, a homeobox transcription factor whose expression is induced by Nkx2.1 (Du et al., 2008), into adulthood, at least at low levels (Figure 1). Thus, in principle, all MGE-derived INs can be targeted using Nkx2.1 or Lhx6 cumulative genetics. BAC transgenic cre driver lines have been generated for both Nkx2.1 (Xu et al., 2008) and Lhx6 (Fogarty et al., 2007). The Nkx2-1(BAC)-Cre driver efficiently labels the vast majority of MGE-derived INs when paired with a cre reporter line (e.g., Ai14), albeit with somewhat less efficiency for SST INs arising from the dorsal MGE, likely due to a genomic positional effect on the expression of the BAC transgene (see “Caveats and other considerations” section below). However, the dorsal MGE can be targeted with an Nkx6.2-CreER driver (Sousa et al., 2009; He et al., 2016). In addition to Nkx2.1(BAC)-Cre, an Nkx2.1-Flpo knock-in line has been generated that efficiently labels all MGE-derived INs when paired with a flp-dependent reporter line (He et al., 2016).

While recent efforts to identify a progenitor marker similar to Nkx2.1 that is selective for the CGE have been promising (Lee et al., 2022), this is still a work in progress; however, the expression of Adarb2 appears to mark all CGE-derived INs in the adult (Figure 1). Broad labeling of CGE INs has been achieved using an Htr3a(BAC)-EGFP transgenic line (DH30Gsat) (Lee et al., 2010; Vucurovic et al., 2010), with the latter being an effective means to distinguish all CGE INs from MGE INs in postnatal animals (Rudy et al., 2011). However, the pan-CGE IN labeling observed in this high copy number BAC transgenic line is a distortion of the endogenous Htr3a expression pattern, which is largely restricted to a CCK+ subset of VIP and Sncg INs in the adult mouse brain (Figure 1) (Ferezou et al., 2002; Machold et al., 2023). This discrepancy likely arises from the widespread but transient nature of Htr3a (5HT3aR) expression in most CGE INs, consistent with a developmental role for 5HT3aR during early migration of CGE INs into the cortex (Murthy et al., 2014). Additional Htr3a driver lines include an Htr3a(BAC)-Cre line (NO152) (Gerfen et al., 2013; Miyoshi et al., 2015) and an Htr3a-Flpo knock-in line (Schuman et al., 2019); the latter driver lines each label a subset of CGE INs in somatosensory barrel field cortex as compared to Htr3a(BAC)-EGFP (Machold et al., 2023) [Htr3a(BAC)-Cre; Ai9: ~90%; Htr3a-Flpo (het); Ai65F: ~30%; Htr3a-Flpo (hom); Ai65F: ~60%]. Other broad markers for CGE INs expressed during development include Prox1 (Rubin and Kessaris, 2013; Miyoshi et al., 2015) and Sp8/Sp9 (Ma et al., 2012; Wei et al., 2019).

## Meis2

INs expressing the homeobox transcription factor Meis2 have not been well characterized to date. These INs likely originate from the lateral ganglionic eminence (LGE), where Meis2 expression is highly expressed in comparison to the MGE (Toresson et al., 1999). Meis2 INs have been described in the olfactory bulb (Allen et al., 2007) and are sparsely present in the cortical white matter (Frazer et al., 2017), along with a heterogeneous population of interstitial INs (von Engelhardt et al., 2011). While a full discussion of the origins and diversity of olfactory bulb INs is beyond the scope of this review, it is worth mentioning that these INs (Meis2 and non-Meis2 subtypes) arise from the LGE during embryogenesis (Stenman et al., 2003) and continue to be generated postnatally from neurogenic niches along the subventricular zone, following which they migrate along the rostral migratory stream to populate the olfactory bulb (Lledo et al., 2008).

## Id2

The population of CGE INs has been recognized for some time to include both VIP+ and non-VIP+ cells, with Reelin (Reln) being a marker for the latter group (Lee et al., 2010; Miyoshi et al., 2010; Rudy et al., 2011; Tremblay et al., 2016). However, since Reln is also expressed in the majority of SST INs (Miyoshi et al., 2010; Pohlkamp et al., 2014), it is not ideal for identifying non-VIP+ CGE INs, particularly in deeper cortical layers. Although less studied, this group represents a significant fraction of the INs in superficial layers: 90% in L1 and 27% in L2/3 (more abundant than SST INs in these layers). The main IN species in this non-VIP+ CGE group is the neurogliaform cell (NGFC), an IN subtype with distinctive spider-like axonal morphology and extensive local output connectivity due to its cloud-like volume release of GABA (Olah et al., 2009; Overstreet-Wadiche and McBain, 2015). NGFCs have been identified in slice preparations by their high levels of NPY expression (Kubota et al., 2011), and through the use of an NPY(BAC)-hrGFP transgenic line (Chittajallu et al., 2013; Neske et al., 2015; Schuman et al., 2019), but as with Reelin, NPY is also expressed in the majority of SST INs (e.g., see Figure 4). Thus, in the absence of a specific marker such as PV, SST, or VIP, NGFC have been overlooked in many IN studies due to the lack of molecular tools to target this unique IN population. Interestingly though, in contrast to other IN subtypes, NGFC INs were found to arise from both Nkx2.1+ (MGE) and non-Nkx2.1+ lineages, with the majority of hippocampal NGFCs being of MGE origin (Tricoire et al., 2011; Pelkey et al., 2017). The embryonic origin of non-Nkx2.1+ NGFCs (the overwhelming majority of cortical NGFCs) has been proposed to be the preoptic area (POA), based on the use of an Nkx5.1(BAC)-Cre transgenic line (Hmx3-iCre) (Gelman et al., 2009). Since the POA is largely derived from Nkx2.1+ progenitors (He et al., 2016), it remains to be determined which non-Nkx2.1+ germinal zone within the POA or ventral CGE territories gives rise to cortical NGFCs. Nevertheless, this Nkx5.1(BAC)-iCre transgenic line has been useful for targeting and characterizing the development of the cortical NGFC population (Niquille et al., 2018; Gomez et al., 2023).

In addition to NGFC, a number of other understudied IN species within the umbrella of non-VIP/non-SST/non-PV INs (i.e., the fourth major group) have been identified and characterized to date. These include a sparse CCK+ basket cell type that was initially described in the hippocampus (Katona et al., 1999; Klausberger and Somogyi, 2008) and studied in the cortex using a GAD65-EGFP transgenic line (Galarreta et al., 2004). Furthermore, studies on the INs residing in cortical layer 1 (L1) have revealed that these cells included NGFCs but also other distinctive IN cell types (Ibrahim et al., 2020; Schuman et al., 2021; Huang et al., 2024). Examination of single-cell transcriptomic data to uncover differentially expressed genes in IN subpopulations has revealed a number of new markers for these IN subtypes, including Lamp5 and Sncg as putative markers for NGFC and CCK basket cells, respectively (Tasic et al., 2016, 2018; Gouwens et al., 2020; Dudok et al., 2021a). Within the Lamp5 population, NDNF was identified as a marker selectively expressed in the majority of L1 INs, including L1 NGFCs (Tasic et al., 2016) and a distinct L1 NDNF IN population given the name canopy cells based on their superficial location and extended horizontal axonal morphology (Schuman et al., 2019). In addition to NDNF INs, the population of L1 INs was also found to include cells with high levels of α7 nAChR (Chrna7) expression (Schuman et al., 2019), a cell type that roughly corresponds to the “single bouquet cell” (Lee et al., 2015; Zhu, 2023). Recently, the gene Id2 was identified as a marker whose expression encompasses the vast majority of INs within this heterogeneous fourth group (Machold et al., 2023).

To a first approximation, Id2 INs represent ~18% of total INs in the cortex and are roughly comprised of NGFC (~80%) and non-NGFC (~20%) subtypes (Machold et al., 2023). Examination of the IN clusters from Yao et al. (2021) reveals the different IN species from the cortex and hippocampus that make up the Id2 IN group (Figure 2). First, within the NGFC INs, there is a fundamental lineage split between those that are MGE-derived, that is, arising from Nkx2.1+ progenitors, as evidenced by residual Lhx6 expression (Figure 2A, bins 5–9), and those that are CGE (or non-Nkx2.1+ POA) derived (Figure 2A, bins 10–12). In the overall IN scatterplot, these Id2/Nkx2.1 (or Lamp5/Lhx6) cells form a distinct cluster away from other NGFCs (Figure 2C). Interestingly, these MGE-derived NGFCs are mostly found within the hippocampus where they comprise the majority of NGFCs, in contrast to the cortex where most NGFCs are CGE/POA derived (Overstreet-Wadiche and McBain, 2015; Yao et al., 2021). These MGE-origin NGFCs have been targeted using intersectional genetics with an Id2-CreER driver line combined with Nkx2.1-Flpo (e.g., Id2-CreER; Nkx2.1-Flpo; Ai65 for tdTomato labeling) and in mouse cortex were found to be a sparse NGFC population located mainly in deep cortical layers (Krienen et al., 2020; Valero et al., 2021). By optotagging these cells (Id2-CreER; Nkx2.1-Flpo; Ai80), they could be identified during silicon probe recordings and were found to exhibit a uniquely anti-correlated activity profile during cortical down states in sleep (Valero et al., 2021). Also of interest is the observation from comparative studies that this IN type is proportionally more abundant in the primate cortex compared to the mouse (Hodge et al., 2019; Krienen et al., 2020).

NGFCs of CGE origin are comprised of two main subtypes: those that express NDNF and are mostly located within L1 (or hippocampal SLM), and those that are non-NDNF that are located in L2-6. The primary NDNF+ NGFC population (bin 11) expresses moderate levels of NPY (bin 10, located mainly in the frontal cortex, has relatively low levels of NPY) but high levels of Reln; this is in contrast to the non-NDNF NGFC population (bin 12) that expresses very high levels of NPY but is only weakly Reln+ (Figure 2A). In addition to the NDNF NGFC INs, there are two other distinct branches of NDNF+ INs (bins 15–18, Figure 2A, and bins 27–29, Figure 2B). The first branch (bins 15–18) likely encompasses the NDNF canopy cell population identified in L1 via the use of an NPY(BAC)-hrGFP transgenic reporter line to distinguish NDNF NGFC from NDNF canopy cells (Schuman et al., 2019). However, endogenous NPY expression level alone does not appear to be sufficient to resolve NDNF IN subtype heterogeneity (Gouwens et al., 2020). Consistent with this, intersectional genetic targeting with NDNF-Flpo and NPY-Cre does not distinguish L1 NGFC from canopy cells (Hartung et al., 2024), likely due to low levels of NPY expression in the latter. Thus, a multifactorial genetic approach is necessary to fully appreciate NDNF IN diversity, especially across species (Chartrand et al., 2023). The second NDNF+ branch (bins 27–29; Figure 2B) is distinguished by the expression of NtnG1 and its selective hippocampal location (HPF), but the identity of these cells is presently unknown. Several NDNF driver lines have been developed for targeting NDNF cells, including NDNF-dgCre (destabilized cre) (Tasic et al., 2016), NDNF-Cre (Schuman et al., 2019), NDNF-CreER, and NDNF-Flpo (Abs et al., 2018).

In addition to NDNF INs, cortical L1 also harbors a smaller population of INs that can be distinguished by their high levels of Chrna7 expression (α7 cells) (Boyle et al., 2011; Schuman et al., 2019). These α7 INs are largely restricted to L1 and express Reln and Cck, but for the most part, do not express NDNF (Figure 2). Interestingly, even within this α7+ IN population, there are two distinct branches, with one expressing the paired box transcription factor Pax6 (bins 19–20), a marker previously identified in a subset of L1 cells (Zeisel et al., 2015). The other α7 branch (bins 13–14) can be distinguished by the expression of Egln3 and Deptor (Yao et al., 2021; Chartrand et al., 2023), two markers that are also expressed in an adjacent canopy cell type (bin 15), perhaps indicative of some molecular continuity between α7 and canopy cell types. Targeting of α7 cells using an existing Chrna7-Cre knock-in driver line (Rogers et al., 2012) or with two separate knock-in lines made by us (Chrna7-CreER and Chrna7-ires-dgCre) was not successful due to misexpression of Cre in all cases, indicating that the endogenous regulation of Chrna7 transcription is particularly sensitive to sequence alterations introduced during driver line construction.

Beyond α7 INs, there exists an astonishing degree of transcriptomic diversity in strongly Cck + INs (Figure 2B), despite their relative sparseness (~3% of total INs). Two main molecular groups of Cck INs have been distinguished by the Allen Institute in their transcriptomic analyses: Serpinf1 and Sncg (Tasic et al., 2018; Yao et al., 2021). Both the Serpinf1 (bins 21–25) and Sncg (bins 31–39) express high levels of Cck and Cnr1, which encodes for the CB1 cannabinoid receptor. These cells likely correspond to the CCK basket cell type identified in the hippocampus (Klausberger and Somogyi, 2008) and cortex (Galarreta et al., 2004) that exhibits the unusual property of DSI (depolarization-induced suppression of inhibition) mediated by retrograde signaling via endocannabinoids released by pyramidal neurons (Wilson and Nicoll, 2001). Intriguingly, a subset of these basket cells express the vesicular glutamate transporter VGLUT3 (Somogyi et al., 2004; Fasano et al., 2017; Pelkey et al., 2020). Recently, a Sncg-Flpo line has been described that enables genetic targeting of these CCK basket cells (Lee et al., 2021; Dudok et al., 2021a). Some of the subtypes within the Serpinf1 and Sncg groups express VIP (bins 21, 34, 36, and 37) and thus would be included in VIP/CCK intersectional targeting (see below).

## VIP

VIP INs comprise a diverse population of CGE-derived cells mostly located in cortical layers 2–4 (Pronneke et al., 2015; Apicella and Marchionni, 2022) that include interneuron-selective (IS) subtypes, that is, INs that mainly target other INs instead of pyramidal neurons. Consistent with this, VIP INs have been shown to play an important role in mediating cortical disinhibition during arousal (e.g., during active exploration), primarily via the inhibition of SST INs (Lee et al., 2013; Pi et al., 2013; Tremblay et al., 2016; Kullander and Topolnik, 2021). While VIP IN subtype diversity has not yet been fully characterized, a fundamental distinction between VIP/CCK and VIP/CR (Calb2) subtypes has emerged. Using intersectional genetic approaches with a VIP-Flpo driver line combined with either CCK-Cre or CR (Calb2)-Cre, VIP/CCK INs were found to include cells with multipolar morphologies that exhibited output to both pyramidal neurons and other INs, whereas VIP/CR cells possessed bipolar morphologies and targeted efferents to INs (mainly Sst) exclusively (He et al., 2016; Paul et al., 2017; Guet-McCreight et al., 2020). Examination of the transcriptomic profile of VIP INs reveals a complex array of subtypes (Figure 3), even within the VIP/CCK subpopulation which as described above includes some INs from the Sncg and Serpinf1 groups (bins 21, 34, 36, and 37) in addition to those within the main VIP group (bins 41, 44, and 52). A comparative transcriptomic analysis of VIP/CCK and VIP/CR subtypes (Paul et al., 2017) with the overall VIP population revealed the existence of a third group of VIP INs that do not express either Cck or Calb2 (Tasic et al., 2018); some of the markers that exhibit overlap with this group include Crh (Harris et al., 2018; Riad et al., 2022), Tac2 and Cxcl14 (Wu et al., 2022), and Pcdh11x (Tasic et al., 2018). Other markers for subsets of VIP INs include Chat (Dudai et al., 2021), Mybpc1 and Parm1 (Jiang et al., 2023), and Nos1 in hippocampal VIP INs (bins 54 and 55). Of note, there is a largely uncharacterized population of Calb2 INs within the VIP group that are weakly or non-VIP expressing (bins 56–62), some of which are marked by Igfbp6 (bins 58–59) (Tasic et al., 2016), underscoring the diversity of Calb2 INs in the cortex (Cauli et al., 2014).

## SST

SST INs as a group exhibit a tremendous amount of transcriptomic diversity (Figure 4), consistent with the emerging evidence for specialized circuit functions of individual SST IN subtypes (Xu et al., 2013; Tremblay et al., 2016; Muñoz et al., 2017; Schwiedrzik and Freiwald, 2017; Green et al., 2023; Hostetler et al., 2023; Wu et al., 2023; Chamberland et al., 2024). In the sensory cortex, in contrast to VIP cells, most SST IN somas are located in deep layers (L5-6) (reviewed in Tremblay et al., 2016). To a first approximation, SST INs can be divided into long range (LR), Martinotti (MC), and non-Martinotti (non-MC) groups that diverge early in development (Fisher et al., 2024), with additional subtypes evident within each category by adulthood (Figure 4). SST LR INs are characterized by extensive axonal projections that can span cortical and even extracortical areas; thus, they are actually GABAergic projection neurons rather than INs *per se* (Caputi et al., 2013). These cells (bins 63–65) express particularly high levels of Nos1 (nNos) and have been targeted using Sst/Nos1 intersectional genetics, pairing Sst-Flpo with a Nos1-CreER driver that with one dose of tamoxifen selects for these strongly Nos1+ SST cells (He et al., 2016). Intriguingly, the transcriptome of SST LR cells is so distinct that they cluster away from the rest of the Sst IN population (Figure 4B). Martinotti SST INs, loosely defined as SST INs that project axons to superficial cortical layers (L1-3) and target the apical dendrites of pyramidal cells (in addition to other INs), can be delineated as T-shaped (most of the ascending axon in L1) or fanning-out (axon in L2/3 and L1), with each type exhibiting distinct activity profiles in somatosensory cortex during whisking behavior (Muñoz et al., 2017). In the hippocampus, the analogous SST population are the oriens-lacunosum moleculare (OLM) INs, which characteristically extend their axons from the oriens to SLM to target pyramidal neuron apical dendrites (Klausberger and Somogyi, 2008; Caroni, 2015).

A number of recent studies have begun to illuminate the diversity of SST MC, non-MC, and OLM IN populations using intersectional genetic approaches (He et al., 2016; Nigro et al., 2018; Gouwens et al., 2020; Wu et al., 2023; Chamberland et al., 2024). However, the insights from earlier work using mouse lines (GIN, X94, and X98) with short promoter GAD67-EGFP transgenic insertions that labeled different subsets of SST INs due to remarkably specific founder effects should not be overlooked as this approach enabled the first characterization of non-MC and MC subtypes (Ma et al., 2006; Xu et al., 2013; Hostetler et al., 2023). Currently, there are a variety of genetic approaches to target SST IN subtypes. A subset of L5 MC with T-shaped morphology and OLM INs can be labeled using a Chrna2(BAC)-Cre line with excellent specificity (Hilscher et al., 2017; Siwani et al., 2018; Hilscher et al., 2023; Chamberland et al., 2024); this population (bins 77–82) includes the SST/Myh8 subtype in cortex (Wu et al., 2023). A distinct T-shaped MC population (bins 66–69) can be targeted by using a Pdyn-CreER driver and excluding SST/NPY+ cells by taking advantage of the cre-dependent reporter Ai9 design, in which Flp activity removes reporter expression (Pdyn-CreER; NPY-Flpo; Ai9) (Wu et al., 2023). Fanning-out MC subtypes can be targeted using intersectional genetics with Sst-Flpo, for example, SST/CR (bin 98), which labels a fanning-out subtype primarily located in L2/3, likely corresponding to the GIN population (Ma et al., 2006; He et al., 2016; Nigro et al., 2018). A distinct deep layer fanning-out subtype can be targeted with SST/Etv1 intersectional genetics (bins 83–87) (Wu et al., 2023). SST non-MC INs are also surprisingly diverse, with Sst/Crh (L5b-6; bins 70–76), Sst/Crhr2 (L6; bins 88–93), and Sst/Hpse (L4; bins 94–97) subtypes (Wu et al., 2023). Of note, there is some degree of continuity between the Sst and Pvalb IN clusters, with a Th + subtype forming a bridge of cells between the two main groups (Figure 4B). Tac1 is a marker for most Pvalb INs (Pfeffer et al., 2013) (Figure 5A), but there is an Sst/Tac1 subtype described recently in the hippocampus that interestingly exhibits a high degree of output selectivity for PV INs vs. pyramidal cells (Chamberland et al., 2024), supporting the idea that in addition to VIP/CR INs, some SST IN subtypes may act in a disinhibitory manner (Xu et al., 2013).

## PV

Despite being the most abundant IN population, PV INs appear to possess less transcriptomic diversity than the other IN groups (Figure 5). However, there is a fundamental division in the PV group between the basket cell (bins 111–121) and axo-axonic cell (AAC) or chandelier cell (ChC; bins 122–123) subtypes, reflected by the distinct clustering of AAC from other PV INs (Figure 5B). Cortical PV basket cells are located across L2-6, where their axons form extensive perisomatic baskets on pyramidal neurons (reviewed in Tremblay et al., 2016). In contrast, cortical PV ChC INs are mostly located in L2, where they extend their axons horizontally to target the axon initial segments of pyramidal neurons (Taniguchi et al., 2013; Inan and Anderson, 2014). Targeting of PV INs as a group can be achieved with Pvalb-Cre or Flp driver lines, or by use of rAAVs with PV IN-specific promoters (e.g., E2) (Vormstein-Schneider et al., 2020). Expression of PV itself in basket cells can vary across cortical areas; for example, most entorhinal/perirhinal fast-spiking basket cells express little to no PV (Nigro et al., 2021). Selective targeting of ChC was first achieved by taking advantage of the distinct developmental trajectories of ChC and PV basket cells. Interestingly, while most MGE INs (including PV basket cells) rapidly downregulate Nkx2.1 expression following their specification, ChC are born relatively late during embryogenesis and also maintain Nkx2.1 expression for a few days during their tangential migration (~E18-P3). Thus, ChC can be targeted using an Nkx2.1-CreER (tamoxifen-inducible cre) knock-in driver to selectively label Nkx2.1+ ChC INs at late embryonic stages (Taniguchi et al., 2011, 2013). To improve experimental access to ChC in adult animals, this developmental Nkx2.1-CreER strategy was paired with a Cre-dependent Flp reporter line (R26-CAG-loxP-stop-loxP-Flpo), thereby resulting in permanent Flp expression in ChC (Lu et al., 2017). In combination with Flp-dependent rAAV injection (rAAV-fDIO-EGFP), this approach enabled the robust labeling and fine reconstruction of individual ChC INs (Wang et al., 2019). More recently, ChC/AAC INs have been successfully targeted with Unc5B-CreER in the hippocampus (Dudok et al., 2021b) and brain-wide using intersectional genetics with Unc5B-CreER; Nkx2.1-Flpo or Pthlh-Flpo; and Nkx2.1-Cre pairing (Raudales et al., 2024).

## Drivers and reporters

Many years of diligent work by numerous laboratories (but particularly the Huang Lab and the Zeng/Allen Institute group) have led to a truly impressive genetic toolkit of driver and reporter lines for targeting IN cell types (Madisen et al., 2010, 2012, 2015; Taniguchi et al., 2011; He et al., 2016; Daigle et al., 2018). A summary of some of the main Cre and Flp driver lines presently available that are relevant for IN studies is shown in Table 1. Along with many other terrific resources provided by the Allen Institute, the transgenic characterization page is highly recommended as it provides an extensive collection of images across the brain of the cumulative cell labeling arising from a variety of driver lines, in addition to the acute expression pattern of each driver^2^ (Harris et al., 2014). Along with the development of driver lines, an extensive array of reporter lines has been established, enabling the conditional expression of a wide range of actuators, including fluorescent proteins, Ca^2+^ activity indicators, engineered channelrhodopsins (ChR), and chemogenetic tools (Table 1). These include the popular Cre-dependent tdTomato reporter lines made by the Allen Institute Ai9 and Ai14 (same reporter as Ai9, except with the neo selection cassette removed) and Ai32 (ChR2/EYFP). For intersectional genetics, building on the pioneering work from the Dymecki Lab (Dymecki et al., 2002; Branda and Dymecki, 2004; Dymecki and Kim, 2007; Jensen and Dymecki, 2014), there is now an expanding range of Cre- and Flp-dependent reporters, including Ai65 (Cre + Flp ➔ tdTomato), Ai80 (Cre + Flp ➔ CatCh ChR), RC::FPSit (Cre + Flp ➔ synaptophysin-YFP), R26-dual-tTA (Cre + Flp ➔ tet activator), RC::FL-hM3Dq (Cre + Flp ➔ Gq DREADD), RC::FL-hM4Di (Cre + Flp ➔ Gi DREADD), and recently developed TIGRE-based lines such as Ai195 (Cre + Flp ➔ GCaMP7s) and Ai211 (Cre + Flp ➔ ChrimsonR ChR). In addition, several intersectional/subtractive reporters have been made that enable dual fluorescent labeling of complementary cell populations: RC::FLTG (Flp ➔ tdTomato; Flp + Cre ➔ EGFP) and IS (Cre ➔ tdTomato; Cre + Flp ➔ EGFP). For viral-based reporters, there is an extensive collection of Cre-dependent (rAAV-DIO or rAAV-flex) or Flp-dependent (rAAV-fDIO) constructs available (e.g., Addgene.org), as well as ongoing innovation in intersectional AAV design (Fenno et al., 2020; Pouchelon et al., 2022; Hughes et al., 2024). Resources for animal husbandry, colony management, and genotyping methods are available from vendors/repositories such as Jackson Laboratories and Taconic Biolabs.

**Table 1:** Driver lines and intersectional reporters.

**Driver line**	**Nature of transgene**	**Jax/MMRRC stock #**	**Reference**	**PMID**
Calb2-ires-Cre	KI	010774	Taniguchi et al. (2011)	21943598
Cck-ires-Cre	KI	012706	Taniguchi et al. (2011)	21943598
Chrna2(BAC)-Cre	BAC tg	MMRRC_036502-UCD	Gerfen et al. (2013)	24360541
Crh-ires-Cre	KI	021704	Taniguchi et al. (2011)	21943598
Crh-ires-Flpo	KI	031559	Salimando et al. (2020)	32277042
Dlx6a-Cre	Tg	008199	Monory et al. (2006)	16908411
Dlx5/6-Flpe	Tg	010815	Miyoshi et al. (2010)	20130169
Gad2-ires-Cre	KI	028867	Taniguchi et al. (2011)	21943598
GIN	Tg	003718	Ma et al. (2006)	16687498
Hmx3(BAC)-Cre	BAC tg	n/a	Gelman et al. (2009)	19625528
Htr3a(BAC)-Cre	BAC tg (NO152Gsat)	MMRRC_036680-UCD	Gerfen et al. (2013)	24360541
Htr3a(BAC)-EGFP	BAC tg (DH30Gsat)	MMRRC_000273-UNC	Lee et al. (2010)	21159951
Htr3a-ires-Flpo	KI	030755	Schuman et al. (2019)	30413647
Id2-CreER	KI	016222	Rawlins et al. (2009)	19855016
Lamp5-P2A-Flpo	KI	037340	n/a	n/a
Ndnf-ires-Cre	KI	030757	Schuman et al. (2019)	30413647
Ndnf-ires-dgCre	KI	028536	Tasic et al. (2016)	26727548
Ndnf-ires-CreERT2	KI	034875	Abs et al. (2018)	30269988
Ndnf-ires-Flpo	KI	034876	Abs et al. (2018)	30269988
Nkx2.1(BAC)-Cre	BAC tg	008661	Xu et al. (2008)	17990269
Nkx2.1-CreER	KI	014552	Taniguchi et al. (2011)	21943598
Nkx2.1-ires-Flpo	KI	028577	He et al. (2016)	27618674
Nos1-CreER	KI	014541	Taniguchi et al. (2011)	21943598
Npy-ires-Cre	KI	027851	Milstein et al. (2015)	26402609
Npy-ires-Flpo	KI	030211	Daigle et al. (2018)	30007418
Npy(BAC)-hrGFP	BAC tg	006417	van den Pol et al. (2009)	19357287
Pvalb-ires-Cre	KI	017320	Hippenmeyer et al. (2005)	15836427
Pvalb-T2A-Cre	KI	012358	Madisen et al. (2010)	20023653
Slc32a1-ires-Cre	KI	028862	Vong et al. (2011)	21745644
Slc32a1-ires-Flpo	KI	029591	Daigle et al. (2018)	30007418
Sncg-ires-Flpo	KI	034424	Lee et al. (2021)	34387544
Sst-ires-Cre	Ki	013044	Taniguchi et al. (2011)	21943598
Sst-ires-Flpo	KI	031629	He et al. (2016)	27618674
Vip-ires-Cre	KI	010908	Taniguchi et al. (2011)	21943598
Vip-ires-Flpo	KI	028578	He et al. (2016)	27618674
X94	Tg	006334	Ma et al. (2006)	16687498

**Intersectional reporter line**	**Genomic locus**	**Jax/MMRRC stock #**	**Reference**	**PMID**
Ai65 (tdTomato)	R26	021875	Madisen et al. (2015)	25741722
Ai80 (CatCh)	R26	025109	Daigle et al. (2018)	30007418
Ai195 (jGCaMP7s)	TIGRE	034112	Allen Institute	n/a
Ai211 (ChrimsonR)	TIGRE	037379	Allen Institute	n/a
RC::FPSit (synaptophysin-YFP)	R26	030206	Niederkofler et al. (2016)	27851959
RC:: FLTG (tdTomato/EGFP)	R26	026932	Plummer et al. (2015)	26586220
IS (tdTomato/EGFP)	R26	028582	He et al. (2016)	27618674
R26-dual-tTA (tTA)	R26	036304	Matho et al. (2021)	34616069
RC::FL-hM4Di	R26	MMRRC 043516-UCD	Lusk et al. (2022)	35086530
RC::FL-hM3Dq	R26	026942	Sciolino et al. (2016)	27264177

## Caveats and other considerations

A fundamental consideration in the use of transgenic targeting strategies is the nature of the transgenic driver line itself, with different caveats to bear in mind when working with short transgenes (e.g., Dlx6a-Cre), BAC transgenic lines (e.g., Htr3a(BAC)-Cre), or knock-in lines. Short transgenes constructed using defined enhancer elements typically less than ~2 kb assemble into multicopy concatemers prior to genomic integration and can exhibit dramatic positional effects on expression that vary from founder to founder depending on the location of the transgene insertion (Palmiter and Brinster, 1986). As described above in the SST IN section, the EGFP expressing transgenic lines X94 and GIN that, respectively, label non-MC and L2/3 MC SST subsets originated from founders harboring Gad67-EGFP transgene insertions subject to remarkably specific but unpredictable positional effects (Ma et al., 2006). Transgenic lines constructed using the Dlx5/6 intergenic enhancer (e.g., Dlx6a-Cre and Dlx5/6-Flpe) benefit from being driven by endogenous Dlx1/2 expression and thus may be more resistant to genomic positional effects; this might account at least in part for the success of rAAV-pDlx constructs (Dimidschstein et al., 2016). BAC transgenic approaches entail the inclusion of typically 50–100 kb of 5′ and 3′ sequence flanking the gene of interest to recapitulate the expression pattern exhibited by the endogenous gene and minimize positional effects (Heintz, 2000); this approach has led to a vast collection of transgenic EGFP and cre driver lines via the GENSAT project (Gong et al., 2003; Gerfen et al., 2013). However, BAC transgenic lines can still exhibit founder effects arising from variation in transgene copy number and the location of genomic insertion, as evidenced within the GENSAT collection^3^ and observed with the Htr3a(BAC)-EGFP and Htr3a(BAC)-Cre lines (Machold et al., 2023). Furthermore, BAC transgenic lines may overexpress genes that are within the BAC genomic region that flanks the gene of interest, for example, there is ectopic Htr3b expression in the Htr3a(BAC)-Cre line (Winterer et al., 2019).

**Figure 6:**
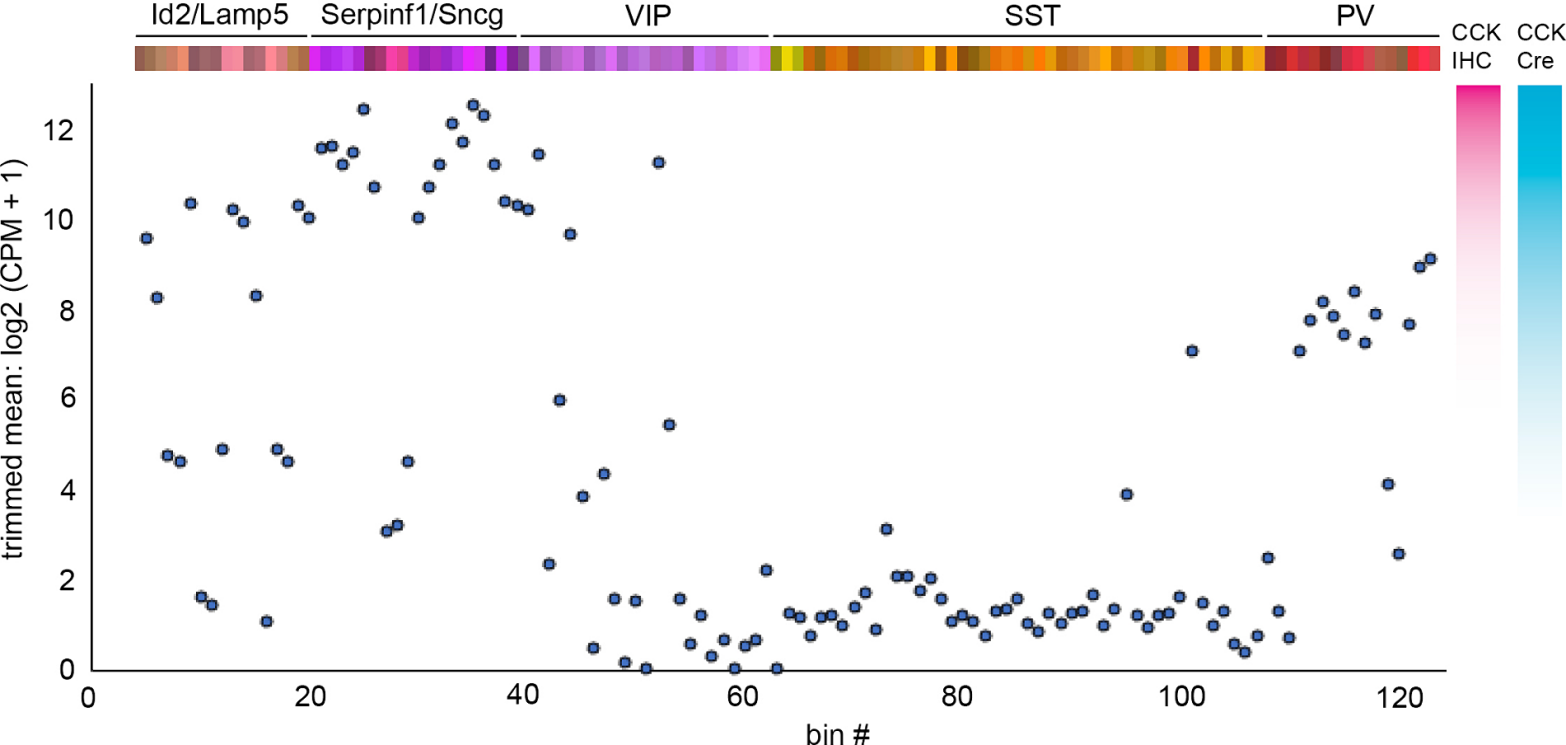
Cck mRNA expression levels across INs. Plot of trimmed mean log2 (CPM + 1) Cck mRNA transcript levels for each IN bin using values from Yao et al. (2021). To the right of the plot are illustrative comparisons of the hypothetical degree of cell labeling using CCK immunohistochemistry (IHC) or Cck-Cre; reporter cumulative genetic labeling. Note that the latter method labels a substantial number of Id2/Lamp5 and PV INs (Machold et al., 2023).

Also of fundamental importance when using driver lines expressing recombinases such as Cre or Flp is the spatiotemporal expression trajectory of the driver gene itself. For many of the commonly used IN driver lines, the cumulative recombination pattern obtained by pairing driver and reporter lines aligns well with the mature expression profile (Harris et al., 2014). However, certain genes may exhibit developmental expression that then results in much broader reporter expression beyond the intended target population (e.g., NDNF; Schuman et al., 2019). In those cases, it is necessary to use Cre- or Flp-dependent viral reporters that can be injected at adult ages (e.g., rAAV-DIO or rAAV-fDIO constructs) or to design driver lines with destabilized cre recombinases (NDNF-dgCre; Tasic et al., 2016) or tamoxifen-inducible CreER (NDNF-CreER; Abs et al., 2018). Furthermore, it is crucial to consider the relative specificity of a driver gene’s expression across different cell types. An example of this is Cck, which is expressed at high levels in a subset of VIP and Sncg/Serpinf1 INs but also at lower levels in NGFC as well as PV cells (Figure 6), resulting in significant labeling of those INs in Cck-Cre intersectional crosses (Machold et al., 2023). Another example of this is the “off-target” labeling of a subset of PV INs in Sst-Cre; Ai14 animals (Hu et al., 2013), which is not an artifact of genetics but instead a reflection of the existence of a small population of PV cells with sufficient Sst expression to drive reporter expression, also evident in the transcriptome data (Figures 4, 5). A critical caveat with both PV and SST drivers is that both genes are expressed at low levels in subsets of pyramidal cells: PV in L5 PCs (Hafner et al., 2019; Palicz et al., 2024) and SST in CA3 hippocampal PCs (Muller-Komorowska et al., 2020). Thus, future studies that target PV or SST INs should strongly consider using intersectional approaches (e.g., by using rAAV-pDlx DIO constructs instead of those with pan-neuronal promoters) to avoid incidental expression in PCs.

It is difficult to predict the level of recombinase expression that will result in a particular reporter being expressed, although in general, cre recombinase is somewhat more efficient than flp (Zhao et al., 2023). The efficiency of recombination of different reporters can vary substantially depending on the loxP and/or frt flanked transcriptional stop cassettes (or DIO/fDIO design); for example, we have observed that the intersectional tdTomato reporter Ai65 is more sensitive and labels more cells when compared with RC::FLTG. A key consideration with any recombinase-conditional or enhancer-dependent approach is the leakiness of the expression system. Genetic tdTomato reporter lines such as Ai9/Ai14 or Ai65 exhibit excellent signal-to-noise ratios (i.e., recombinase-dependent expression vs. leakiness), but a low level of background expression that is difficult to detect with fluorescent proteins may be more consequential with reporters expressing sensitive actuators such as other recombinases. In contrast to genetic reporter lines which are typically present as single copies, viral reporters are usually injected at high titer, with multiplicity of infection rates >10^3^ depending on the distance from the injection site. Both rAAV-DIO and rAAV-fDIO constructs have been found to exhibit some leakiness depending on the viral preparation and other variables (Fischer et al., 2019; Botterill et al., 2021); thus, it is essential to include control injections in non-transgenic animals (i.e., not expressing recombinase) to assess off-target expression. Finally, while rAAVs engineered with cell type-specific promoters and/or enhancers show great promise, leakiness and specificity issues must be carefully evaluated. For example, the CamK2 promoter that is widely used for pyramidal neuron targeting in rAAV constructs has recently been shown to exhibit some leaky expression in INs (Veres et al., 2023). As observed previously with short enhancer-based transgenes (Hostetler et al., 2023), short promoters in rAAV constructs (e.g., pSst44; Hrvatin et al., 2019) may display a remarkable degree of specificity for subpopulations of INs that exhibit shared circuit properties (Green et al., 2023) but may not drive expression in the entire IN group being targeted (in this case, SST INs).

An important consideration, perhaps of a more philosophical nature, is how to think about cell type diversity and its relationship to transcriptomic variation. Overall, there appears to be some degree of transcriptomic continuity across certain cortical and hippocampal IN subtypes (Harris et al., 2018; Scala et al., 2021; Yao et al., 2021), supporting the idea that the specific genetic identity of individual INs within a subtype is influenced by local context or even stochastic events during their developmental trajectory and circuit integration. While the transcriptomic properties of each IN likely underlie their morphological and electrophysiological attributes to a large extent, the relationship is complex (e.g., Paul et al., 2017; Gouwens et al., 2020), and there are many additional variables that could be considered when defining an IN “cell type” (Petilla Interneuron Nomenclature Group et al., 2008; Zeng, 2022; Mao and Staiger, 2024). Fundamental attributes of an IN include its input/output organization, which is determined in large part by the morphology and location of its dendrites and axons, but also by molecular interactions between a diverse assortment of cell surface and secreted proteins that begin during early development (Honig and Shapiro, 2020; Sanes and Zipursky, 2020). Many different types of proteins can contribute to an IN’s responsiveness to a specific input in addition to the ion channels and other cell surface molecules that regulate intrinsic membrane properties, including those that participate in downstream intracellular signaling pathways, or protein trafficking to specific cellular compartments. Last but not least, the engagement of a specific IN population during a particular behavioral context can be strongly influenced by the expression of receptors for neuromodulators such as acetylcholine, norepinephrine, and serotonin, or for peptidergic signaling (e.g., oxytocin). Thus, when delineating IN cell subtypes, it ultimately comes down to deciding which attributes to prioritize and which are the most critical in determining the cell’s functional role in brain circuitry.

Why has evolution favored inhibitory IN subtype diversity? The most straightforward explanation lies in the increasingly complex structure of neocortical pyramidal neurons from mice to humans, particularly of their distinctive apical dendrites whose branching complexity has expanded in tandem with the superficial cortical layers (L1-3) that support cortical–cortical connectivity (Schmidt and Polleux, 2021; Galakhova et al., 2022). Considering that even mouse pyramidal neurons each receive on the order of thousands of excitatory inputs, the specialization of GABAergic inputs to different pyramidal neuron compartments and other IN subtypes allows for enhanced control over the integration of information streams arriving at the apical dendrites and soma/basal dendrites [top-down and bottom-up inputs, respectively; reviewed in Schuman et al., 2021]. With regard to human health, there is an emerging consensus that dysfunction of specific IN subtypes may contribute to a wide range of neurocognitive disorders (Ferguson and Gao, 2018; Gallo et al., 2020; Goff and Goldberg, 2021; Yang et al., 2022), including autism (Lunden et al., 2019; Contractor et al., 2021), epilepsy (Jiang et al., 2016), schizophrenia (Dienel and Lewis, 2019), depression (Fogaca and Duman, 2019), and Alzheimer’s disease (Hernandez-Frausto et al., 2023). Furthermore, the elaborate developmental trajectory of INs may lead them to be particularly susceptible to environmental perturbations (Pai et al., 2023) or toxic insults (Ansen-Wilson and Lipinski, 2017). It is our hope that a deeper understanding of the marvelous diversity of INs will help illuminate the inner workings of the brain and facilitate the elucidation of novel therapeutic approaches for treating neurological diseases.

## Author contributions

RM: Conceptualization, Data curation, Visualization, Writing – original draft, Writing – review & editing. BR: Funding acquisition, Resources, Supervision, Writing – review & editing.

## Footnotes

^1^ portal.brain-map.org/atlases-and-data/rnaseq

^2^ connectivity.brain-map.org/transgenic

^3^gensat.org

## Glossary

AAC: Axo-axonic cell
Ai: Allen Institute
BAC: Bacterial artificial chromosome
Calb2/CR: Calbindin-2 (calretinin)
CamK2: Ca2+/calmodulin-dependent protein kinase 2
Cck: Cholecystokinin
CGE: Caudal ganglionic eminence
ChC: Chandelier cell
Chrna2: Cholinergic receptor nicotinic alpha 2 subunit
Crh: Corticotropin-releasing hormone
DIO: Double-floxed (loxP) inverse ORF
Dlx: Distal-less homeobox
DREADD: Designer receptors exclusively activated by designer drugs
fDIO: Double-flrted (frt) inverse ORF
Gad2: Glutamate decarboxylase 2
Htr3a: 5-hydroxytryptamine receptor 3a
Id2: Inhibitor of DNA binding 2
IN: GABAergic interneuron
KI: Knock-in
Lamp5: Lysosomal associated membrane protein family member 5
L1: Layer 1
LR: Long range
Ndnf: Neuron-derived neurotrophic factor
NGFC: Neurogliaform cell
Nkx2.1: Nk2 homeobox 1
Nos1: Nitric oxide synthase 1
NPY: Neuropeptide Y
MC: Martinotti cell
MGE: Medial ganglionic eminence
OLM: Oriens-lacunosum moleculare
ORF: Open reading frame
PMID: PubMed identifier
POA: Preoptic area
Pvalb/PV: Parvalbumin
rAAV: Recombinant adeno-associated virus
Slc32a1: Solute carrier family 32 member 1
Sncg: Synuclein gamma
Sst: Somatostatin
Tg: Transgenic
Vip: Vasoactive intestinal peptide
